# Strengthening surveillance and response to public health emergencies in the West African sub-region: the role of Ghana FELTP

**DOI:** 10.11604/pamj.supp.2016.25.1.10579

**Published:** 2016-10-01

**Authors:** Donne Kofi Ameme, Kofi Mensah Nyarko, Ernest Kenu, Edwin Andrews Afari

**Affiliations:** 1 Ghana Field Epidemiology and Laboratory Training Program (GFELTP), School of Public Health, University of Ghana, P.O. Box LG 13, Accra, Ghana; 2 Ghana Health Service, Accra, Ghana; 3 Department of Epidemiology and Disease Control, School of Public Health, University of Ghana, Legon, Ghana

**Keywords:** Surveillance, public health emergencies, FELTP, Ghana

## Introduction

Effective disease detection, investigation, control, and prevention require a public health workforce well trained in the principles and practice of Field Epidemiology. Since 1980, the United States (U.S) Centers for Disease Control and Prevention (CDC) has worked with Ministries of Health throughout the world to establish and support the establishment of Field Epidemiology and Laboratory Training Programs (FELTPs). The Ghana Field Epidemiology and Laboratory Training Program (GFELTP) established with support from the CDC started implementing its two-year advanced long course in 2007. Ghana FELTP a member of the founding member countries of the African Field Epidemiology Network (AFENET) was the first program to be established in the West African sub-region and also the first to have veterinarians as part the team of medical and laboratory scientist as trainees (one-health approach).

In the wake of emerging and re-emerging diseases that continue to shake the foundations of national response capacities, key competencies needed by the world include the ability to conduct surveillance and use the information for action [1]. These competencies are hinged on a well-trained and well-motivated workforce capable of undertaking such actions. Field Epidemiology Training Programs (FETPs) akin to the Epidemic Intelligence Service program run by the CDC provides the needed workforce. Field Epidemiology workforce development is therefore central to building sustainable health systems for disease prevention and control. Globally, these training programs have emerged as the new wave for preparing a workforce for the next public health emergency.

### The Ghana Field Epidemiology and Laboratory Training Program

The GFELTP has throughout its existence spearheaded the training of Field Epidemiologists in Ghana [2] and beyond. At the core of its training is development of human capital with competencies in public health surveillance, outbreak investigation and response. From a solely national training program, the GFELTP has over the years evolved into a robust sub-regional resource center for training Field Epidemiologists; extending its frontiers to English speaking West African countries such as the Gambia, Liberia and Sierra Leone.

In the face of the need for workforce development for responding to public health problems at the various levels of the heath care system, the full complement of the pyramidal model of FETP training becomes a necessity. The three-tiered “pyramid” model of training addresses the need to improve the surveillance, epidemiology, response, and scientific communication skills of public health workers at each level of the health system. Within each tier, the training focuses on improving the participants’ skills within the context of their current job responsibilities and expectations. The response to this is the implementation of the FETP frontline program in addition to the 2-year advanced program. With its tenth cohort of advanced and seventh cohort of frontline training, the GFELTP has trained over 200 healthcare workers including physicians, nurses, veterinarians, laboratory scientists and disease control officers at all levels of the health sector. The numbers, albeit inadequate, is contributing to a pool of highly skilled human resource, which is rapidly becoming the game changer for surveillance and outbreak investigation and response in Ghana and beyond.

Trainees and alumni have contributed to development at the national and subnational levels by supporting in evaluating surveillance systems, analyzing surveillance data for action, conducting planned epidemiological studies, investigating and responding to public health emergencies (outbreaks in human and animal health). The outputs from these competencies have been widely disseminated to stakeholders at national and international conferences and in peer-reviewed journals. On the international landscape, alumni of the program are serving at key positions within their various ministries and playing leadership roles in supporting national surveillance systems and Field Epidemiology workforce development in other countries.

The leadership contributions from residents and alumni of the program is a tribute to the competencies acquired during training and their ability to serve as change agents in their various places of work. These contributions are done within the context of existing surveillance structures at the national and subnational levels thereby strengthening and enhancing the capacity of existing systems in surveillance and outbreak investigation and response.

In the face of the growing field epidemiology workforce crisis in Africa and given the recent devastating effect of the Ebola outbreak in West Africa, the way forward for the GFELTP is clearly cut out. The need to produce a critical mass of competent Field Epidemiologist at all levels of the health sector becomes all the more pressing. There is the need to increase capacity to train a lot more healthcare workers who will perform topnotch surveillance functions and respond swiftly to public health emergencies. To achieve this feat, implementation of the full scale three-tiered pyramidal model of FETP [3] is the way to go. With the GFELTP currently serving as a sub-regional hub for Field Epidemiology training for Anglophone West Africa, it is imperative that there is a collaboration between it and the other two programs in the sub-region: the West Africa FELTP which is the training center for the Francophone West Africa and the Nigeria FELTP for joint planning and production of the required public health workforce for the sub-region. In addition, sustainable financing and innovation stand out as the key pillars should the narrative for response to public health threats and emergencies change from the status quo. With these in place, the outlook for the future can only be bright.

### The supplement

This supplement highlights a few of the achievements of the GFELTP in terms of transforming the various works done by the residents and alumni into documents for sharing with the scientific community. It is a compilation of some of the outputs that were developed into manuscripts during one of the GFELTP’s training workshops organized for its residents and alumni. The papers in the supplement illustrate the capacity development efforts of the program and highlight the competencies acquired by the residents while in training. The authors have described various works they have done and their contribution to public health practice. These, and the description of the capacity development activities catalogued shows evidence of the GFELTP’s role in strengthening surveillance and response through the building of a competent health workforce. The expectation is that this supplement will serve as a testimonial of achievements of the program and a springboard for the residents to effectively communicate their experiences to the world through peer-reviewed journals.
